# Energy Efficiency Analysis of MIMO Wideband RF Front-End Receivers

**DOI:** 10.3390/s20247070

**Published:** 2020-12-10

**Authors:** Eduil Nascimento Junior, Guilherme Theis, Edson Leonardo dos Santos, André Augusto Mariano, Glauber Brante, Richard Demo Souza, Thierry Taris

**Affiliations:** 1 Group of Integrated Circuits and Systems (GICS), Department of Electrical Engineering, Federal University of Paraná (UFPR), Curitiba 81531-980, Brazil; edson_l@ufpr.br (E.L.d.S.); mariano@ufpr.br (A.A.M.); 2 Department of Electrical Engineering, Eindhoven University of Technology (TU/e), 5612 Eindhoven, The Netherlands; g.theis@tue.nl; 3 Department of Electrical Engineering, Federal University of Technology (UTFPR), Curitiba 80230-901, Brazil; gbrante@utfpr.edu.br; 4 Department of Electrical Engineering, Federal University of Santa Catarina (UFSC), Florianopolis 88040-900, Brazil; richard.demo@ufsc.br; 5 IMS Laboratory, University of Bordeaux, 33405 Talence, France; thierry.taris@ims-bordeaux.fr

**Keywords:** wideband RF front-end receiver, LNA, LNTA, energy efficiency

## Abstract

Energy-efficiency is crucial for modern radio-frequency (RF) receivers dedicated to Internet of Things applications. Energy-efficiency enhancements could be achieved by lowering the power consumption of integrated circuits, using antenna diversity or even with an association of both strategies. This paper compares two wideband RF front-end architectures, based on conventional low-noise amplifiers (LNA) and low-noise transconductance amplifiers (LNTA) with N-path filters, operating with three transmission schemes: single antenna, antenna selection and singular value decomposition beamforming. Our results show that the energy-efficiency behavior varies depending on the required communication link conditions, distance between nodes and metrics from the front-end receivers. For short-range scenarios, LNA presents the best performance in terms of energy-efficiency mainly due to its very low power consumption. With the increasing of the communication distance, the very low noise figure provided by N-path LNTA-based architectures outperforms the power consumption issue, yielding higher energy-efficiency for all transmission schemes. In addition, the selected front-end architecture depends on the number of active antennas at the receiver. Hence, we can observe that low noise figure is more important with a few active antennas at the receiver, while low power consumption becomes more important when the number of active RF chains at the receiver increases.

## 1. Introduction

A very relevant use case in wireless communication systems presently is the Internet of Things (IoT) [1]. Moreover, with the fifth-generation (5G) technology, the market is expanding towards massive IoT deployments connected to complex smart sensor networks, which require low-cost devices with low power consumption [2]. Since IoT devices typically consist of battery-powered nodes, energy efficiency (EE) is a critical issue, so that long-term operation without battery replacement is feasible. This demand makes EE a key design goal for 5G IoT applications [3].

Multiple antenna (MIMO) systems have been used to mitigate the effects of fading, which allows improving the link reliability so that wireless nodes transmit with reduced power. However, each antenna should be connected to a radio frequency (RF) chain and, therefore, multiple antennas may increase the power consumption at the circuit level. In that sense, antenna selection (AS) is an important technique [4], yielding the same diversity gains as MIMO, but with lower power consumption due to the reduced number of active RF chains. For instance, the EE optimization of some MIMO techniques, when considering the effect of reconfigurable RF transceivers, is discussed in [5]. Along with AS, singular value decomposition (SVD) beamforming and single antenna (SISO) are considered. Results show that EE can be improved considerably with the AS technique for short and moderate distances. On the other hand, the spatial diversity gains provided by SVD are important to extend the range. In addition, the combination of antenna selection and low-resolution phase shifters for device-to-device communications is investigated by [6]. The proposed antenna selection algorithm is shown to increase the energy efficiency by suppressing interference from other devices in the network, as well as improving the received signal power.

Another important venue to improve the EE is to reduce the power consumption at the circuit level, e.g., through battery management techniques [7] or receiver architecture redesigns [8]. CMOS technology downscaling imposes huge challenges for RF designers, which need to meet severe system requirements while keeping power-consumption as low as possible. Seeking for low-power front-end receivers compliant with the required specifications of 5G-based IoT scenarios, both receiver architecture and circuits have to be optimized. To support a huge number of applications in different communication standards, usually multiple RF front-ends are designed to operate in a single band/standard each, thus potentially consuming considerable power overall [9]. Hence, a low-power wideband RF front-end receiver is essential for multi-standard communications.

**Figure 1:**
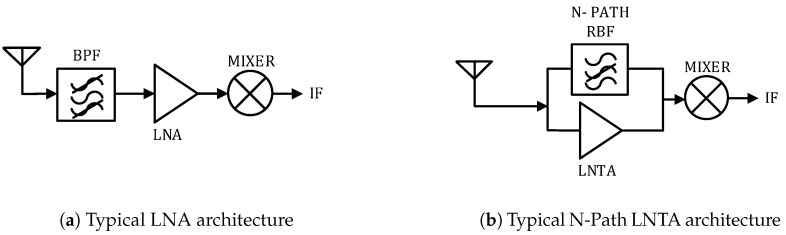
LNA and LNTA front-end receiver architectures for wideband operation.

Figure 1 illustrates two different wideband receiver architectures. The conventional receiver chain in Figure 1a has a band-pass filter (BPF), followed by a low-noise amplifier (LNA) and an active mixer. In wideband applications, BPF are generally surface acoustic wave (SAW)-filters organized in banks, which are switched on/off depending on the band or channel required. These SAW-filters make the receiver bulky and costly. Several strategies on the LNA designs can be found in the literature for wideband receivers, depending on the metric to be optimized. To save die area, wideband inductorless LNAs with tunable active shunt-feedback architecture have been proposed in [10]. Also employing an active shunt-feedback, but focusing on reducing noise figure, a wideband noise-canceling CMOS LNA with enhanced linearity was proposed by [11]. In addition, aiming at reducing the power-consumption and improving the linearity, the circuit designed in [12] is a dual-path noise and nonlinearity canceling LNA. Furthermore, instead of designing a single wideband LNA, a multi-standard beamforming RF front-end is presented in [13], which consists of four independent receiving paths. This circuit covers a wideband operation, yielding better noise figure and linearity at the expense of increasing the overall power-consumption.

Common to the previously mentioned low-power wideband LNAs is their focus on providing low noise figure at the expense of reduced gain and linearity. Alternatively, Figure 1b presents the low-noise transconductance amplifier (LNTA) architecture. The multiple off-chip bulky SAW-filters are replaced by on-chip reconfigurable filters, denoted as N-path filters [14]. Then, the combination of the high output impedance of LNTA with the low impedance of the N-path circuitry enables significant attenuation of frequencies outside the desired band, thus providing high RF selectivity on-chip. Therefore, the association of the LNTA with the multiple reject-band filters (RBF) in parallel allow the reception and amplification of the desired signal in wideband, through multiple smaller bands, allowing reduced noise figure at the front-end. In addition, to reduce power-consumption, a passive mixer is placed right-after the LNTA. The position of the RBF could change around the LNTA, depending on the desired behavior. LNTAs have recently drawn considerable interest due to their greater linearity performance [15].

For instance, inductorless designs implementing noise and distortion cancellation techniques are presented in [16,17]. Moreover, a high-linearity RF receiver architecture is shown in [14], adopting Miller band-pass filters for channel selection. To reduce the front-end receiver power consumption, in [18] the LNTA is followed by a current mode passive mixer that provides sufficient linearity to permit coexistence with large out-of-band interference arising from other transceivers. Nevertheless, despite the enhanced linearity of LNTAs, these circuits face severe requirements for impedance matching networks and bandpass filters. As a result, occupied area enlarges significantly. In addition, to improve receiver sensitivity, LNTAs are designed to provide a sufficient gain to the weak incoming RF signals, increasing the power consumption [19]. In summary, the above mentioned LNTA-based designs aim to improve linearity and reduce the noise figure, at the expense of a significant increase in the power consumption.

Given all the challenges imposed by energy-constrained applications, in this paper we present an energy efficiency analysis that merges aspects of front-end receiver architectures with the spatial diversity improvements of using multiple antennas. Then, we take into account the power consumption of the required circuit blocks, for both LNA and LNTA architectures, in two MIMO scenarios: one based on AS scheme, in which only one pair of antennas remains active in each transmission; and other based on SVD beamforming, which uses all transmitting and receiving antennas to increase robustness against channel fading. Moreover, as a reference, we also consider the SISO case. Results show that low power consumption is not enough to guarantee the best performance in terms of energy efficiency. There is a tradeoff between link distance, noise figure and power consumption that can be optimized through the proper combination of receiver architectures and spatial diversity techniques.

The remainder of this paper is organized as follows. Section 2 details the RF receiver designs and the communication model. The considered transmission schemes are mathematically detailed in Section 3. Then, some numerical results are discussed in Section 4, while Section 5 concludes the paper.

## 2. Preliminaries

### 2.1. Receiver Circuit Designs

Figure 1 illustrates the typical blocks for the LNA and LNTA architecture. In the figure, only the front-end part of the receiver is considered, i.e., until the intermediate frequency (IF). Seeking for a more realistic communication model, we selected some GHz band design examples from the state-of-the-art. The main features of these silicon-proven RF circuits are included in our model, according to Table 1. It is worth noting that our methodology for choosing the wideband RF front-end receivers is based on the power consumption and noise figure performance results presented by the circuit designs. Moreover, we have chosen three RF circuits for each architecture presenting similar receiver sensitivity.

Common to all architectures is the fact that the receiver circuit designer works to optimize the RF front-end, with a special focus on the first block of the receiver chain, since it directly affects the receiver sensitivity and noise figure. The receiver sensitivity is the lowest received signal power at the antenna, for which the signal can be correctly decoded, and can be calculated (in dBm) as

(1) $$S_{\mathrm{rx}}=N_{0}\;\left[\mathrm{dBm}\right]+F_{\mathrm{rx}}\;\left[\mathrm{dB}\right]+10\;\log_{10}\left(B\right)+\xi_{0}\;\left[\mathrm{dB}\right],$$

where $N_{0}=kT$ is the thermal noise power spectral density, in W/Hz, with *k* being the Boltzmann’s constant and T=290 K the temperature, *B* is the channel bandwidth and $\xi_{0}$ is the minimum signal-to-noise ratio (SNR) required for correct decoding. Moreover, $F_{\mathrm{rx}}$ is the cascaded noise figure of the receiver. In the nomenclature for circuit design, the noise figure is expressed in dB, while the noise factor is its linear form. Nevertheless, for simplicity, throughout this paper we will employ only the term noise figure, which is given by the Friis formula [20]

(2) $$\begin{array}{cc} F_{\mathrm{rx}} & =F_{\mathrm{FirstBlock}}+\frac{F_{\mathrm{SubsequentBlocks}}-1}{G_{\mathrm{FirstBlock}}}\\ \; & \approx F_{\mathrm{FirstBlock}}, \end{array}$$

where $F_{\mathrm{FirstBlock}}$ is the noise figure of the first block, which depends on the RF front-end receiver architecture, $F_{\mathrm{SubsequentBlocks}}$ is the combined noise figure of the subsequent blocks, which is divided by the power gain of the first block $G_{\mathrm{FirstBlock}}$. In addition, the total noise figure mainly depends on the first block, and the approximation is valid since $G_{\mathrm{FirstBlock}}$ is usually high, according to Table 1. Also, we consider that the RF receivers are operating in their linear region and interference is implicitly handled by an upper layer multiple access protocol, so that any residual interference can be accounted for as additional noise in our system model.

**Figure 2:**
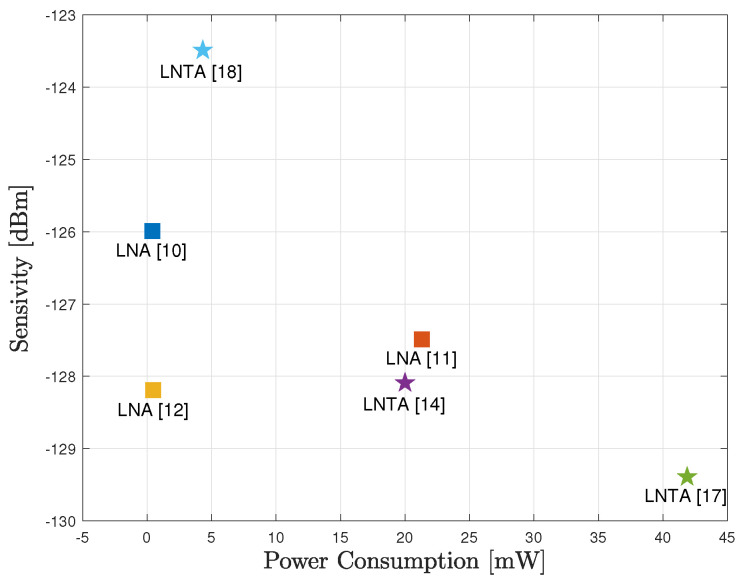
Sensitivity as a function of the power consumption of the RF front-end architectures, with B=20 MHz and $\xi_{0}=0$ dB.

Figure 2 illustrates the sensitivity of the RF circuits shown in Table 1 as a function of the power consumption, considering B=20 MHz and $\xi_{0}=0$ dB. As we observe, small sensitivity can be obtained with both architectures, e.g., considering the LNA design from [12] and the LNTA from [17]. However, the advantage of improved receiver sensitivity is not always evident to maximize the EE, so that small power consumption may be of greater importance and the LNTA from [18] may also be interesting design choice. Such situation may be of particular interest when the communication distance increases, so that the transmitted power becomes much larger compared to the power consumption of the circuits. As a consequence, the relative advantages and disadvantages of each RF circuit design will be evaluated in order to maximize the EE.

### 2.2. Communication Model

**Figure 3:**
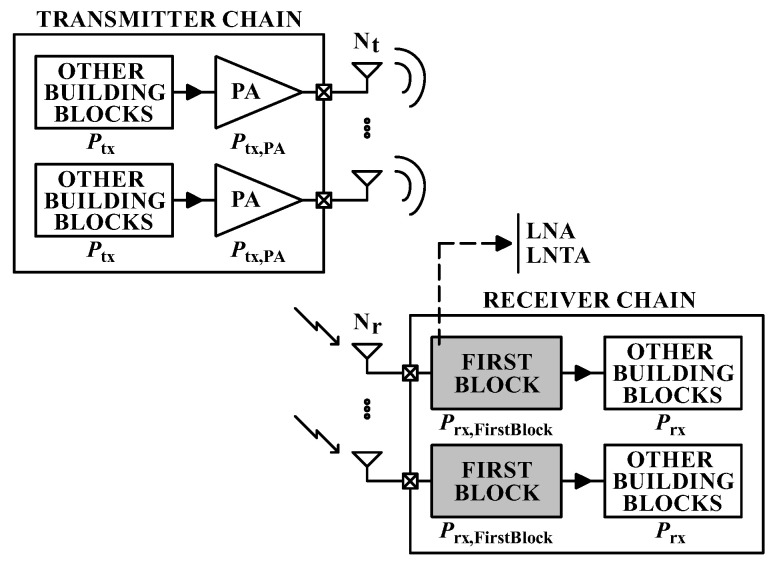
RF transceiver building blocks.

We consider a system as illustrated in Figure 3, with a transmitter node and a receiver node, in which we use $N_{t}$ and $N_{r}$ to represent the number of available transmitting and receiving antennas, respectively; while lower case letters $n_{t}$ and $n_{r}$ denote the active number of antennas at each node. In addition, each antenna is assembled on a single RF chain.

Besides the power used to transmit the RF signal, the consumption of all building blocks at the transmitter and receiver nodes needs to be included in order to achieve the total system power budget. Thus, considering that $n_{t}\leq N_{t}$ RF chains are active at the transmitter and $n_{r}\leq N_{r}$ RF chains are active at the receiver, the total power consumption becomes

(3) $$P_{\mathrm{total}}^{\left(\mathrm{sch}\right)}=n_{t}\left(P_{\mathrm{tx},\mathrm{PA}}+P_{\mathrm{tx}}\right)+n_{r}\left(P_{\mathrm{rx},\mathrm{FirstBlock}}+P_{\mathrm{rx}}\right),$$

where the subscript sch∈{SISO,AS,SVD} indicates the transmission scheme, which we detail in Section 3,

(4) $$P_{\mathrm{tx},\mathrm{PA}}=P_{\mathrm{out}}/\eta$$

is the power amplifier (PA) consumption, with $P_{\mathrm{out}}$ being the transmitted power that the PA forwards to the transmitter output, and η represents the PA drain efficiency [21]. It is worth remarking that in the case of multiple active RF chains, we assume that the transmitted power is equally distributed among all PAs. Furthermore, $P_{\mathrm{rx},\mathrm{FirstBlock}}$ is the power consumed by the first block at the receiver chain, depending on the employed LNA or LNTA architecture, according to Table 1. In addition, $P_{\mathrm{tx}}$ and $P_{\mathrm{rx}}$ are the power consumption of the other building blocks at the transmitter and receiver, respectively.

Then, a communication link connecting the transmitter and receiver nodes can be expressed as

(5) $$y=\sqrt{\kappa \;P_{\mathrm{out}}}\;H\;x+w,$$

where y is the received signal vector with dimensions $N_{r}\times 1$, κ is the link budget relationship, H is the channel fading matrix of size $N_{r}\times N_{t}$, whose elements $h_{ij}\in H$, ∀i,j are independent and identically distributed random variables, whose envelops follow aNakagami-*m* distribution. Moreover, x is the $N_{t}\times 1$ unit energy transmitted symbol vector and w is a $N_{r}\times 1$ vector of additive white Gaussian noise (AWGN) with variance $N_{0}/2$ per dimension.

The link budget relationship is given by [22]

(6) $$\kappa =\frac{G_{a}\;\lambda^{2}}{{\left(4\pi \right)}^{2}\;d^{\alpha }\;M_{l}},$$

where $G_{a}$ is the product of the transmitter and receiver antenna gains, $\lambda =\frac{c}{f_{c}}$ is the wavelength, $c=3\times 10^{8}$ m/s is the speed of light, $f_{c}$ is the carrier frequency, *d* is the distance between the communicating nodes, α is the path loss exponent and $M_{l}$ is the link margin.

Furthermore, the instantaneous SNR at the receiver is

(7) $$\xi ={∥H∥}_{F}^{2}\cdot \bar{\xi },$$

where ${∥.∥}_{F}$ is the Frobenius norm and the average SNR at each receive antenna with respect to each transmit antenna is given by

(8) $$\bar{\xi }=\frac{P_{r}}{N_{0}\;B\;F_{\mathrm{rx}}},$$

in which $P_{r}=\frac{\kappa \;P_{\mathrm{out}}}{n_{t}}$ is the received power. In addition, it is worth noting that $F_{\mathrm{rx}}$ affects the average SNR at the receiver and, therefore, the EE of the communication system.

## 3. Transmission Schemes

In this section, we describe the employed transmission schemes, SISO, AS and SVD beamforming, in terms of their outage probability and energy efficiency expressions.

### 3.1. Outage Probability and Transmitted Power

An outage event in a communication link occurs whenever the received SNR falls bellow a threshold $\xi_{0}$ that allows correct decoding. In other words, the outage probability is defined as $\mathrm{Pr}\left\{\bar{\xi }<\xi_{0}\right\}$, where assuming the use of capacity achieving error correcting codes, $\xi_{0}=2^{R}-1$ and *R* is the spectral efficiency in bit/s/Hz [22].

#### 3.1.1. SISO

We first assume the SISO scheme, where the transmitter and receiver are equipped with only one antenna at each node. Then, the outage probability is [23]

(9) $$O_{\mathrm{SISO}}=\frac{\gamma \left(\frac{\xi_{0}}{\bar{\xi }},m\right)}{\Gamma \left(m\right)}$$

where *m* is the Nakagami-*m* fading parameter, γ(·,·) is the lower incomplete gamma function ([24], Section 6.5.2) and Γ(·) is the complete gamma function ([24], Section 6.1.1).

With that in hand, we assume that the system operates with a given target outage probability $O^{⋆}$, so that $O_{\mathrm{sch}}=O^{⋆}$ is used to find the minimal required transmitted power $P_{\mathrm{out}}$ of each scheme, as in [25]. Then, in the case of the SISO scheme the transmitted power must be adjusted according to

(10) $$P_{\mathrm{out}}^{\left(\mathrm{SISO}\right)}=\frac{\xi_{0}\;N_{0}\;B\;F_{\mathrm{rx}}}{\kappa \;\gamma^{-1}\left(\Gamma \left(m\right)O^{⋆},m\right)},$$

where $\gamma^{-1}\left(\cdot ,\cdot \right)$ is the inverse gamma function, and which depends on the communication distance, as well as on the employed receiver architecture. Furthermore, let us remark that $P_{\mathrm{out}}^{\left(\mathrm{sch}\right)}$ represents the power used with the LNTA architectures, while there is an additional term due to the SAW-filters of the LNA architectures, as it will be detailed in Section 3.2.

#### 3.1.2. AS

In this case only one antenna is active at both transmitter and receiver. The pair of antennas can be chosen during the transmission of pilot symbols prior to each frame, in which the best antenna at the receiver is selected based on the highest received SNR, whereas the best antenna at the transmitter is selected via a feedback channel. The outage probability of AS is given by [23,25]

(11) $$O_{\mathrm{AS}}={\left[\frac{\gamma \left(\frac{\xi_{0}}{\bar{\xi }},m\right)}{\Gamma \left(m\right)}\right]}^{N_{t}N_{r}},$$

yielding the following required transmitted power

(12) $$P_{\mathrm{out}}^{\left(\mathrm{AS}\right)}=\frac{\xi_{0}\;N_{0}\;B\;F_{\mathrm{rx}}}{\kappa \;\gamma^{-1}\left(\Gamma \left(m\right){\left(O^{⋆}\right)}^{\frac{1}{N_{t}N_{r}}},m\right)}.$$

#### 3.1.3. SVD

In this scheme, all antennas are used at the transmitter and receiver nodes, which increases the robustness to channel fading, but also increases the power consumption due to the number of active RF chains. In addition, a feedback channel is also required for SVD; however, the capacity of the feedback channel must be much higher than in the case of AS. Following [23,25], the outage probability of SVD is

(13) $$O_{\mathrm{SVD}}\approx \frac{\gamma \left(\frac{\rho_{0}}{\bar{\xi }},mN_{t}N_{r}\right)}{\Gamma \left(mN_{t}N_{r}\right)},$$

where $\rho_{0}=n\left(2^{R/n}-1\right)$ and $n=\min\{N_{t},N_{r}\}$.

Then, the transmitted power of the SVD scheme

(14) $$P_{\mathrm{out}}^{\left(\mathrm{SVD}\right)}=\frac{\rho_{0}\;N_{0}\;B\;F_{\mathrm{rx}}}{\kappa \;\gamma^{-1}\left(\Gamma \left(mN_{t}N_{r}\right)O^{⋆},mN_{t}N_{r}\right)}.$$

### 3.2. Effect of the SAW-Filters at the LNA Receiver

Another important element that affects the transmitted power is the SAW-filter at the LNA receiver. For the LNA architecture we consider the insertion loss of the off-chip SAW-filters and the necessary arrangements for the transmission of the simultaneous carriers. For the LNTA architecture this was not necessary because integrated reconfigurable filters are implemented. As a result, there is a 2.1 dB loss, per active receive antenna, in the received SNR of the LNA, compared to the LNTA. The value of 2.1 dB is extracted from the datasheet of a commercial low insertion loss RF SAW filter [26], compliant with the carrier frequency denoted in Table 2.

Then, in order to provide the same SNR $\xi_{0}$ required to meet the target outage probability $O^{⋆}$ for both architectures, we compensate such insertion loss of the LNA by increasing the transmitted power with respect to that required with the LNTA architecture, i.e.,

(15) $$P_{\mathrm{out},\mathrm{LNA}}^{\left(\mathrm{sch}\right)}\;\left[\mathrm{dBm}\right]=P_{\mathrm{out}}^{\left(\mathrm{sch}\right)}\;\left[\mathrm{dBm}\right]+n_{r}\times 2.1\;\left[\mathrm{dB}\right].$$

### 3.3. Energy Efficiency

We define the EE, in bit/J/Hz, as

(16) $$\eta_{\mathrm{sch}}=\frac{R\cdot \left(1-O_{\mathrm{sch}}\right)}{P_{\mathrm{total}}^{\left(\mathrm{sch}\right)}},$$

in which the numerator represents the system throughput, in bit/s/Hz, and $P_{\mathrm{total}}^{\left(\mathrm{sch}\right)}$ is the total power consumption of each scheme. Let us recall that, according to (3), $P_{\mathrm{total}}^{\left(\mathrm{sch}\right)}$ depends on the number of active antennas of each transmission scheme, with $n_{t}=n_{r}=1$ for both SISO and AS schemes, while $n_{t}=N_{t}$ and $n_{r}=N_{r}$ for the SVD scheme. In addition, the power consumption of the circuit blocks also depends on the employed RF front-end architecture.

It is also worth noting that AS is expected to yield a higher EE than SISO, since the outage probability of AS is lower than that of SISO, for the same total power consumption. On the other hand, the outage probability of the SVD scheme is lower than AS; however, since all antennas are active in both transmitter and receiver, the power consumption is also higher, which leads to a trade-off in terms of EE.

## 4. Results and Discussions

In this section we provide a few numerical results with the two considered RF front-end architectures. The system parameters are listed in Table 2. In addition, we consider nodes from a wireless sensor network with the same number of antennas to transmit and receive, $N_{t}=N_{r}$. The transmission system is evaluated using the closed-form expressions for the outage probabilities. In other words, given the target outage probability $O^{⋆}$ we find the required transmission power for each scheme, using (10), (12) and (14), respectively for the SISO, AS and SVD schemes. In addition, these expressions also depend on the noise figure of the receiver, so that each RF front end architecture impacts the required minimum transmission power. With that in hand, the energy efficiency of each scheme is computed using (16).

Table 3 shows the energy efficiency of the LNA designs, selected from [10,11,12], as a function of the transmission distance. Then, for each LNA we consider SISO, AS and SVD schemes, with the architecture that yields the best performance highlighted in orange, cyan and green shadings, respectively. Please note that the front-end designs with the lowest power consumption are not necessarily the ones with the best energy efficiency. The association of low noise figure and low power consumption clearly plays a very important role in designing an energy efficient system. Then, in Table 3 we observe that the LNA in [12] yields the highest energy efficiency for SISO, AS and SVD. Next, Table 4 considers the LNTA designs selected from [14,17,18]. As we observe, the LNTA in [14] yields the highest energy efficiency for SISO when d=50 m, being outperformed by the LNTA in [17] when d≥100 m. Using the AS scheme, the LNTA in [18] performs best when d=50 m, the LNTA in [14] performs best when d∈{100,150} m and the LNTA in [17] performs best when d≥200 m. Therefore, the blue shading shows the highest energy efficiency across architectures. Finally, the SVD scheme follows the same idea, so that the best architecture starts with the LNTA in [18] when d≤150 m, shifting to the LNTA in [14] when 200m≤d≤350 m and to the LNTA in [17] when d≥400 m. As a consequence, in the sequel we pick the LNA/LNTA architectures with the best performance among their counterparts. According to Table 3, the LNA in [12] is the best choice, while the LNTA in [17] outperforms the other LNTAs most of the time according to Table 4.

**Figure 4:**
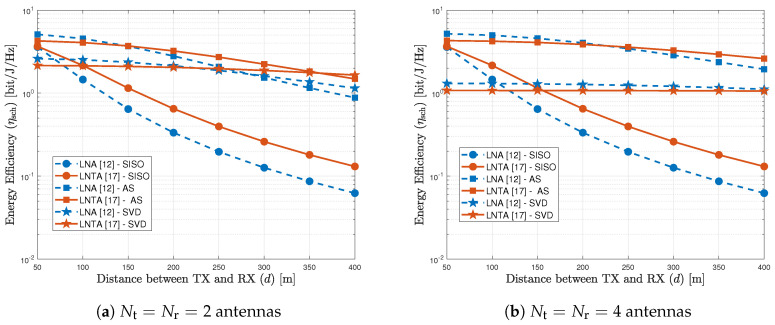
Energy efficiency of SISO, AS and SVD schemes, considering the LNA [12] and LNTA [17] architectures.

Figure 4a plots the energy efficiency as a function of the distance between transmitter and receiver considering the LNA in [12] and the LNTA in [17], for the SISO, AS and SVD schemes with $N_{t}=N_{r}=2$ antennas. As we can observe, the LNTA architecture at the receiver usually achieves higher energy efficiency, except for short transmission ranges with the MIMO schemes. In this example, the LNA outperforms the LNTA in terms of EE with AS when d≤150 m, while this distance increases up to d≤220 m with the SVD scheme. This indicates that the very low power consumption of the LNA design in [12] plays an important role to maximize the EE in short transmission distances, while the lower noise figure of the LNTA from [17] becomes more important when the distance increases, since it allows alleviating the transmitted power of the PA, at the transmitter side. Complementing the analysis, the importance of the spatial diversity brought by the multiple antennas becomes more evident in Figure 4b, which increases the number of antennas to $N_{t}=N_{r}=4$. As we observe, similar conclusions can be drawn for the AS scheme, where the LNA becomes more energy efficiency for up to d≤220 m. However, when submitted to the SVD scheme, the LNA presents better performance than the LNTA. The performance difference decreases when the transmission distance increases.

**Figure 5:**
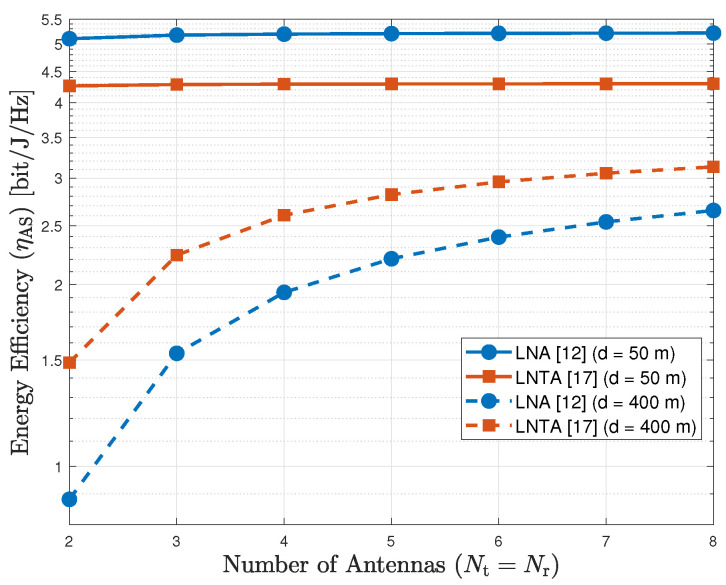
Energy efficiency of the AS scheme as a function of the number of antennas ($N_{t}=N_{r}$), for d={50,400} m.

Next, Figure 5 shows the energy efficiency of the AS scheme as a function of the number of antennas, considering $N_{t}=N_{r}$ and d={50,400} m, for the LNA and LNTA architectures. As we observe, when d=50 m the LNA outperforms the LNTA in terms of energy efficiency, while this conclusion inverts for d=400 m. As previously mentioned, the noise figure of the selected LNTA is lower than that of the LNA counterpart, which becomes more important when the distance increases, regardless of the number of antennas. In addition, we also observe that the energy efficiency increases with the number of antennas up to a saturation level.

**Figure 6:**
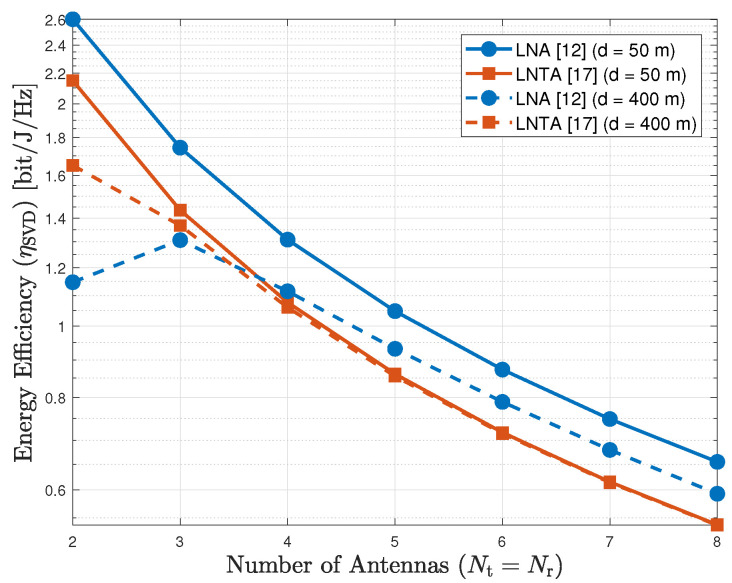
Energy efficiency of the SVD scheme as a function of the number of antennas ($N_{t}=N_{r}$), for d={50,400} m.

For the SVD scheme, on the other hand, there is an optimal number of antennas to maximize the energy efficiency, which depends on the transmission distance, as depicted by Figure 6 for d={50,400} m. Similarly as for the AS scheme, the LNA outperforms the LNTA for short transmission distances. When *d* increases, the LNTA performs better for a reduced number of antennas (less than 4). Improving the spacial diversity allows the LNA to surpass the LNTA, even presenting a worst noise figure. For the SVD scheme, the number of antennas determines the number of receivers. Hence, power consumption becomes a key metric in the energy efficiency performance.

**Figure 7:**
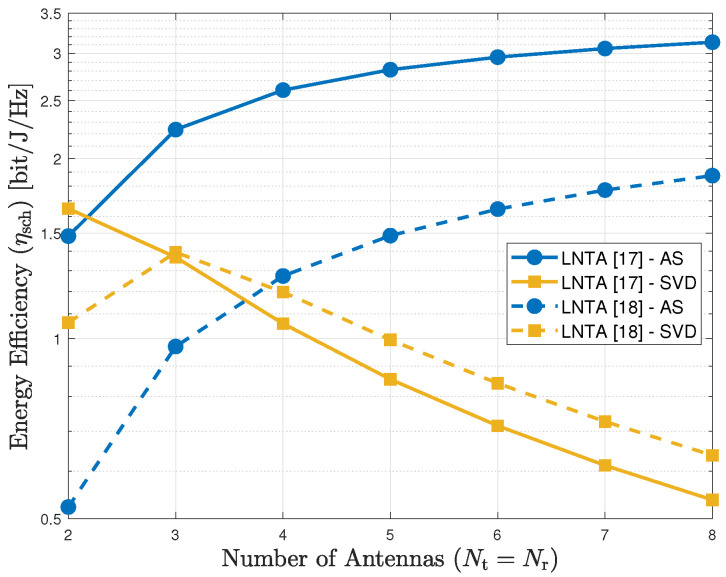
Energy efficiency of the AS and SVD schemes as a function of the number of antennas for the LNTA designs in [17,18], for d=400 m.

Finally, Figure 7 plots the energy efficiency of the AS and SVD schemes as a function of $N_{t}=N_{r}$, comparing the LNTAs from [17] and [18], for d=400 m. As we observe, the LNTA designed in [17] is always more energy efficient with the AS scheme, in which a single pair of RF chains is active for communication. However, there is a trade-off between [17] and [18] for the SVD scheme when the number of antennas increases. With a few antennas (less than 3), the energy efficiency using [17] is higher, while it is outperformed by [18] with more antennas. By taking the parameters of Table 1 into account, we observe that the LNTA in [17] has very low noise figure and high power consumption, with $F_{\mathrm{rx}}=1.6$ dB and $P_{\mathrm{rx},\mathrm{FirstBlock}}=41.88$ mW. On the other hand, the design in [18] has very low power consumption at the expense of a higher noise figure, with $F_{\mathrm{rx}}=7.5$ dB and $P_{\mathrm{rx},\mathrm{FirstBlock}}=4.32$ mW in this case. Therefore, we observe that low noise figure is more important when the number of active antennas is low (e.g., with AS and SVD with a few antennas), while low power consumption becomes crucial when the number of active RF chains at the receiver increases.

## 5. Conclusions

Energy efficiency behavior modeling has been presented in this paper. The proposed model sought to place different front-end receivers in relation to different communication schemes. More precisely, two different receiver architectures composed by LNA and LNTA, respectively, were employed with SISO, AS, and SVD schemes, for different ranges.Nakagami-*m* fading distribution is used to characterize the wireless channel, in order to be better compliant with IoT scenarios, since the parameter *m* can be used to model scenarios where nodes have line-of-sight conditions. After an analysis of the energy efficiency between receivers based on the state of the art, the best candidate for each front-end architecture was selected. A comparative performance study of the different communication schemes was carried out, showing which distances and conditions stand out for the selected front-end designs. The results show that, for short-range scenarios, LNA presents increased EE performance, particularly due to its very low power consumption. On the other hand, when the communication distance increases the very low noise figure provided by N-path LNTA-based architectures outperforms the low power consumption of the LNA-based designs, yielding higher EE for SISO and AS transmission schemes. For SVD, the LNA always presents the better performance in terms of EE, exposing that IoT applications are strongly dependent on energy consumption of the RF circuits. Finally, our analysis also shows that the selected front-end architecture depends on the number of active antennas at the receiver, so that low noise figure is more important with a few active antennas at the receiver, while low power consumption becomes more important when the number of active RF chains at the receiver increases.

## Figures and Tables

**Table 1:** Characteristics of the selected LNA and LNTA-based receivers.

Architecture	Ref.	Freq. Range [GHz]	Gain [dB]	Power Consump. [mW]	Noise Figure [dB]
**LNA**	[10]	0.1–2.2	12.3	0.4	5
	[11]	0.1–2	17.5	21.3	3.5
	[12]	2.3–2.5	17.4	0.48	2.8
**LNTA**	[14]	0.05–2.5	38	20	2.9
	[17]	0.7–3.8	47	41.88	1.6
	[18]	2–2.8	43.4	4.32	7.5

**Table 2:** Simulation parameters.

Parameter	Description	Value
*B*	Channel bandwidth	20 MHz
$M_{l}$	Link margin	20 dB
$G_{a}$	Total antenna gain	5 dBi
$f_{c}$	Carrier frequency	2.4 GHz
$N_{0}$	Noise PSD	−174 dBm/Hz
α	Path loss exponent	2.5
$O^{⋆}$	Target outage probability	$10^{-3}$
*R*	Spectral efficiency	1 bit/s/Hz
$P_{\mathrm{tx}}$	Power consumption at the TX	97.9 mW [27]
$P_{\mathrm{rx}}$	Power consumption at the RX	92.2 mW [27]
η	Drain efficiency of the PA	0.35% [27]
*m*	Nakagami-*m* fading parameter	2

**Table 3:** Energy efficiency, in bit/J/Hz, as a function of the distance for the different LNA architectures and transmission schemes, with $N_{t}=N_{r}=2$.

Architecture	Scheme	Distance							
		50 m	100 m	150 m	200 m	250 m	300 m	350 m	400 m
**LNA [10]**	SISO	2.9760	0.9874	0.4071	0.2066	0.1203	0.0769	0.0525	0.0377
	AS	5.0165	4.1730	3.0716	2.1387	1.4829	1.0486	0.7620	0.5692
	SVD	2.5915	2.4581	2.2148	1.9036	1.5802	1.2853	1.0368	0.8364
**LNA [11]**	SISO	3.1797	1.2601	0.5508	0.2854	0.1677	0.1077	0.0738	0.0531
	AS	4.5927	4.0607	3.2563	2.4533	1.8050	1.3302	0.9943	0.7573
	SVD	2.3452	2.2664	2.1148	1.9043	1.6632	1.4204	1.1961	1.0003
**LNA [12]**	SISO	3.5927	1.4574	0.6427	0.3341	0.1966	0.1264	0.0866	0.0623
	AS	5.1025	4.5400	3.6759	2.7964	2.0738	1.5373	1.1539	0.8814
	SVD	2.6025	2.5197	2.3596	2.1355	1.8760	1.6115	1.3644	1.1466

**Table 4:** Energy efficiency, in bit/J/Hz, as a function of the distance for the different LNTA architectures and transmission schemes, with $N_{t}=N_{r}=2$.

Architecture	Scheme	Distance							
		50 m	100 m	150 m	200 m	250 m	300 m	350 m	400 m
**LNTA [14]**	SISO	3.7656	1.9126	0.9332	0.5055	0.3031	0.1968	0.1356	0.0979
	AS	4.6817	4.3684	3.8228	3.1688	2.5368	1.9985	1.5704	1.2410
	SVD	2.3715	2.3442	2.2880	2.2009	2.0852	1.9470	1.7943	1.6353
**LNTA [17]**	SISO	3.6607	2.1556	1.1485	0.6481	0.3965	0.2601	0.1804	0.1308
	AS	4.2617	4.0649	3.7006	3.2233	2.7135	2.2359	1.8237	1.4845
	SVD	2.1496	2.1329	2.0982	2.0432	1.9680	1.8749	1.7675	1.6504
**LNTA [18]**	SISO	2.8252	0.9124	0.3733	0.1889	0.1099	0.0702	0.0480	0.0344
	AS	4.8995	4.0277	2.9197	2.0072	1.3794	0.9697	0.7020	0.5230
	SVD	2.5493	2.4604	2.2902	2.0553	1.7882	1.5211	1.2764	1.0641
